# Supplementary material for: pan-ASLM: Axially Swept Light Sheet Microscopy for Fast and High-Resolution Imaging of Expanded Samples
Source: Npj Imaging. 2026 Mar 23;4:16. doi: 10.1038/s44303-026-00141-2 (PMC13009474; doi:10.1038/s44303-026-00141-2)
Supplement: Supplementary file 1 — Supplementary information [file 44303_2026_141_MOESM1_ESM.pdf]

Supplementary Information

pan-ASLM: Axially Swept Light Sheet Microscopy for Fast and High-resolution Imaging of Expanded Samples

Hannahmariam T. Mekbib[1,2], Lasse Pærgård Andersen[2,3], Shuwen Zhang[2], Jonathan Gulcicek[4], Yuan Tian[2], Jack R. Ross[5], Mark D. Lessard[2], Joerg Bewersdorf[1,2,5,6,7]

[1]Department of Biomedical Engineering, New Haven, CT, USA
[2]Department of Cell Biology, Yale School of Medicine, New Haven, CT, USA
[3]DTU Health Tech, Technical University of Denmark, Kgs Lyngby, Denmark
[4]panluminate Inc., New Haven, CT, USA
[5]Department of Physics, Yale University, New Haven, CT, USA
[6]Nanobiology Institute, Yale University, West Haven, CT, USA
[7]Kavli Institute for Neuroscience, Yale School of Medicine, New Haven, CT, USA

Correspondence: joerg.bewersdorf@yale.edu

Supplementary Note 1

Voice coil calibration for high-speed imaging

In pan-ASLM, we use a voice coil actuator for axial sweeping of the light sheet. Replacing the cylindrical lens by a spherical lens forms a beam focus that can be used to visualize the synchronization between the voice coil actuator and the rolling shutter of the camera. Sending a sawtooth waveform to the voice coil, without using the rolling shutter mode, results in an axially uniform pattern that forms across the field of view (**Suppl. Figure 2a**), showing that the remote focusing allows for translation-invariant focus quality. However, in the rolling shutter mode, the line profile degrades as the axial sweeping and the rolling shutter get out of synch for most of the voice coil travel range. This shows that a careful calibration of the voice coil is necessary to achieve synchronization between axially sweeping the light sheet and the rolling shutter of the camera.

To calibrate the voice coil, we first generate a list of voltage values within the range of the FOV and acquire images for the beam focus corresponding to each voltage value. We use Picasso [1] to fit a gaussian function to the brightest pixel position in each image. For a set image acquisition time, we plot the position of the voice coil as a function of time, and we obtain a calibration curve after converting it into a voltage-over-time function with which the voice coil can be controlled (**Suppl. Figure 2b**). We next characterize the calibration curve by fitting a third-order polynomial function. After this calibration, we observe a line profile mostly uniform in the axial sweeping direction across the field of view. However, at the bottom of the field of view, corresponding to the start of the sweeping motion, a ringing pattern can be observed. This ringing can be eliminated by adding flyback time to the function controlling the voice coil motion. This calibration method is suitable for low scan rates of 1 Hz.

To enable higher scan rates, we implemented an automated calibration routine for the voice coil scanner using a global optimizer. The global optimization algorithm minimizes an error function, $f(x)$ by finding x, which is subject to boundary constraints min ≤ x ≤ max set by the user. Briefly, based on the user inputs of minimum and maximum boundaries for each polynomial coefficient used to model the calibration curve, the algorithm sends a signal to the voice coil and calculates an error based on the line profile. The error is calculated by first binning the image (2x16) and then cropping the image to 200x200 pixels. We then fit a gaussian function to each row of pixels and calculate the FWHM (**Suppl. Figure 3a**). The sharper the line, the smaller the FWHM. The mean of the FWHM distribution for each image (line profile) can be calculated and used as an error value corresponding to each line profile. The polynomial coefficients are updated until a lower error (mean) value is achieved, which represents a

uniform line profile across the field of view. In the present setup, the scanning speed in pan-ASLM is limited by the available laser power (as lower exposure times require higher signal) and by the flyback time as we need to allow some time for the voice coil to equilibrate before subsequent scanning. **Suppl. Table 2** provides an overview of the net acquisition times and pixel exposure times for different frame rates.

Supplementary Note 2

pan-ASLM imaging volume and sample thickness

Based on the working distance of the detection objective and the sample chamber size, pan-ASLM is able to image a volume of ~10 × ~15 × ~2 mm^3 (XYZ; **Suppl. Figure 6**). However, this volume is reduced if the sample is less than ~8 mm thick in the Z direction (the direction of the optical axis of the detection objective) since in that case the sample holder clips the light sheet when imaging the edge of the volume that is furthest from both objectives (**Suppl. Figure 13**).

References

[1] Strauss, M. T. (2022). Picasso-server: a community-based, open-source processing framework for super-resolution data. *Communications Biology*, *5*(1), 930.

Supplementary Figures

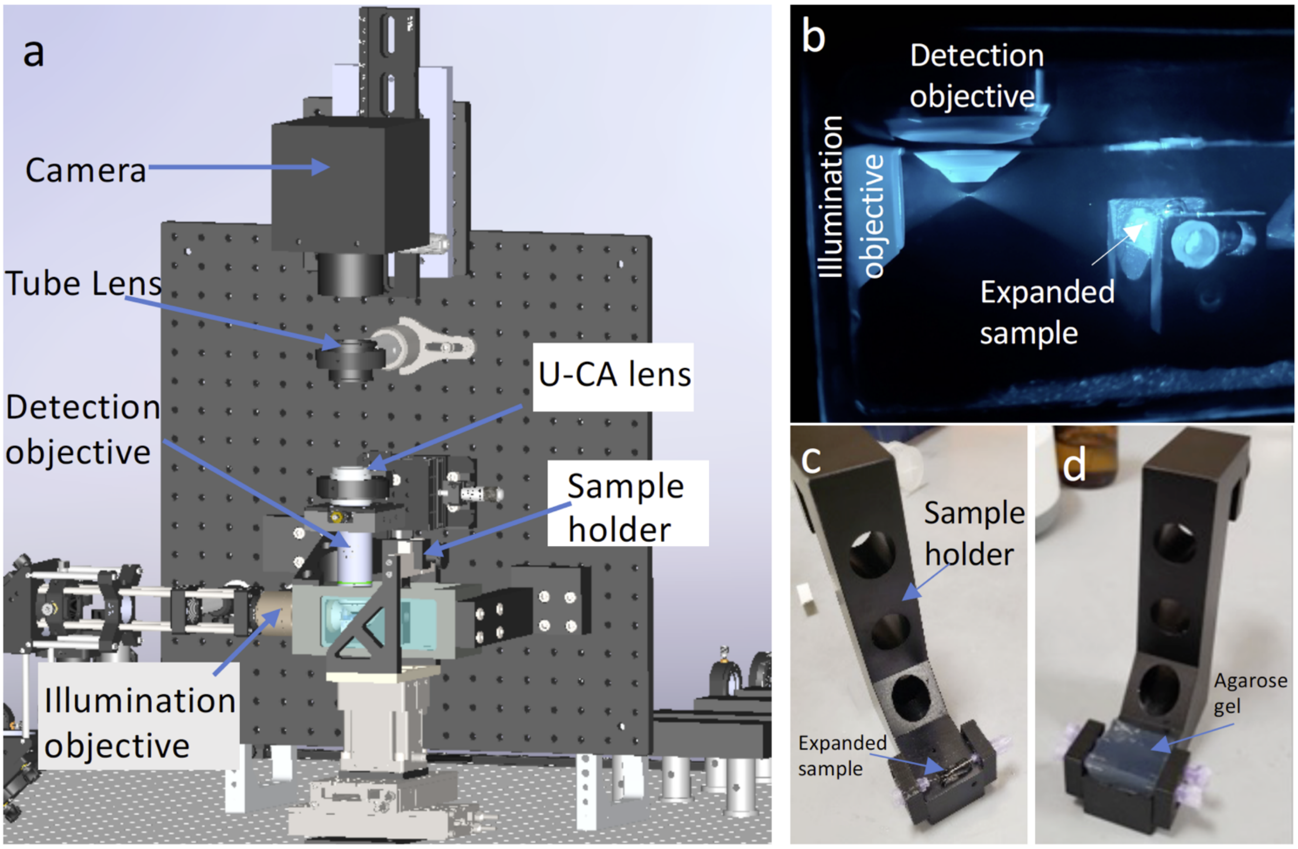

Supplementary Figure 1: pan-ASLM microscope details. a CAD design of the vertical breadboard of the pan-ASLM setup illustrating the detection beam path. **b** Image of the light sheet focused by the illumination objective before an imaging session. **c** Expanded sample mounted on the sample holder. **d** Agarose gel mounted on the sample holder.

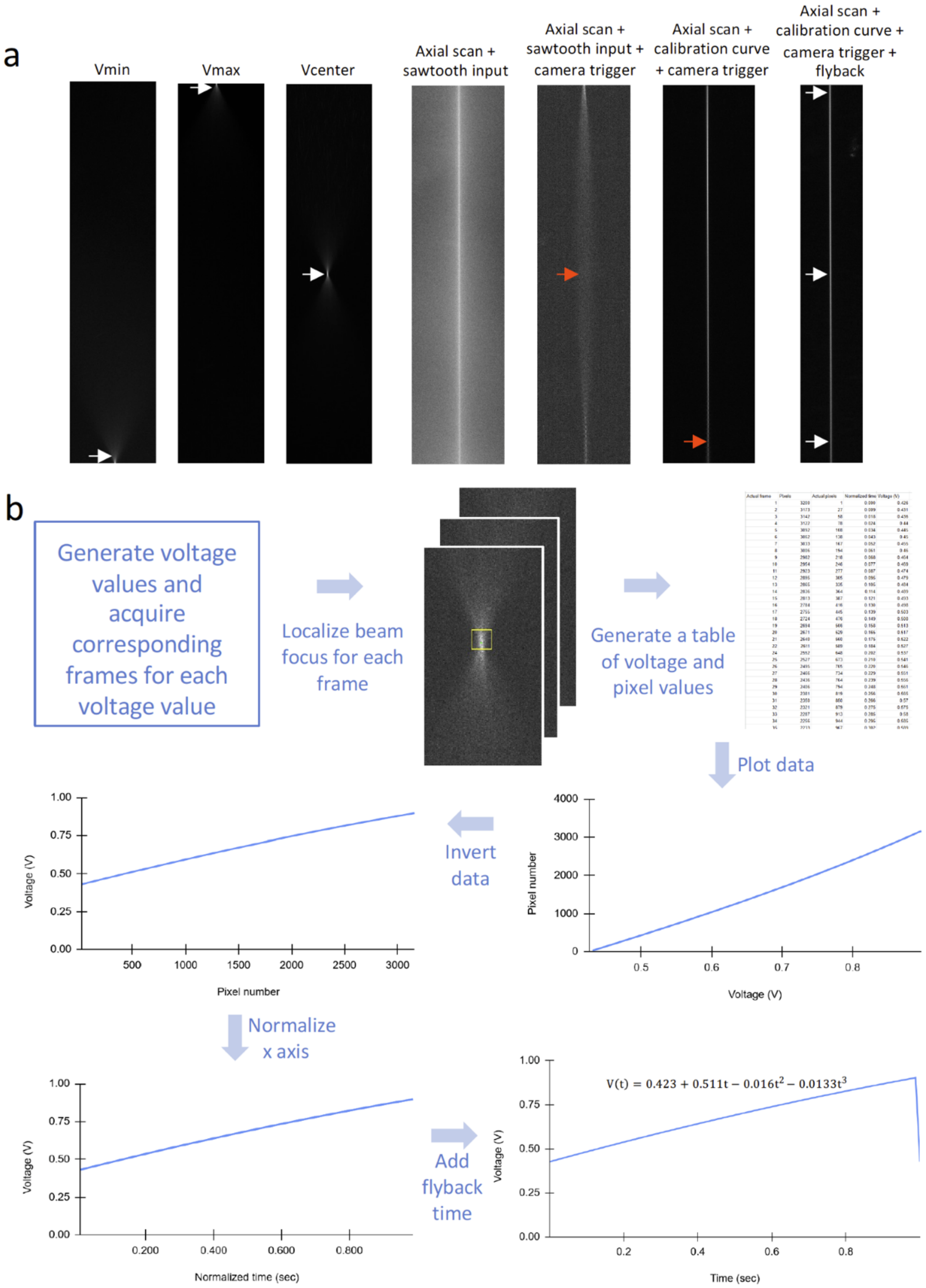

Supplementary Figure 2: Calibration of voice coil actuator for 1 fps frame rate. a (from left to right) Beam focus position corresponding to different voltage values, V_{min}, V_{max} and V_{center}, axial scan without the rolling shutter effect, axial scan with sawtooth voltage input and camera trigger illustrating the lack of synchronization between the voice coil motion and the rolling shutter, axial scan with calibration showing nearly uniform line across the FOV except for a ringing pattern at the bottom of the image, same with flyback time added to the calibration curve resulting in a uniform line across the complete scan range. **b** Process of calibrating the voice coil actuator for 1-Hz scan rate.

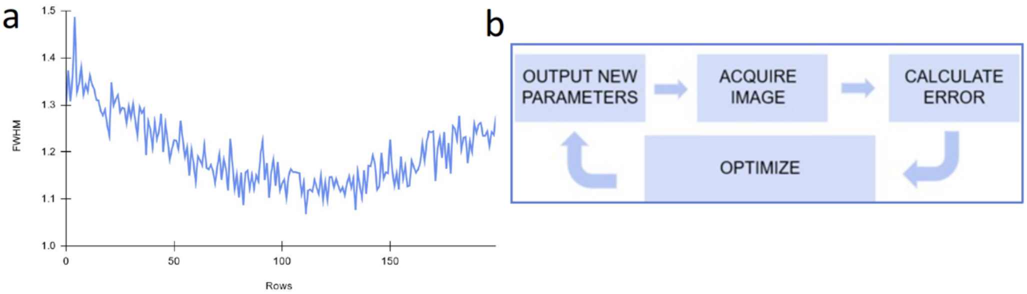

c

Frame rate	Polynomial function representing calibration curve	FOV (pixels)	Voxel rate (Megavoxels/sec)
1 fps	$0.423+0.511t-0.0160t^2-0.0133t^3$	3200 x3200	10.2
5 fps	$0.429+0.530t-0.0160t^2-0.0133t^3$	3200x3155	50.5
10 fps	$0.434+0.567t-0.0163t^2-0.0137t^3$	3200x3117	99.7
20 fps	$0.468+0.578t-0.0178t^2-0.0145t^3$	3200x2860	183

Supplementary Figure 3: Automated calibration routine of voice coil actuator for higher speeds. a FWHM vs. pixel rows graph used to determine uniformity of line profiles across a FOV. **b** Process of automated calibration of the voice coil for higher frame rates. **c** Table showing polynomial functions representing calibration curves for different frame rates and corresponding FOVs.

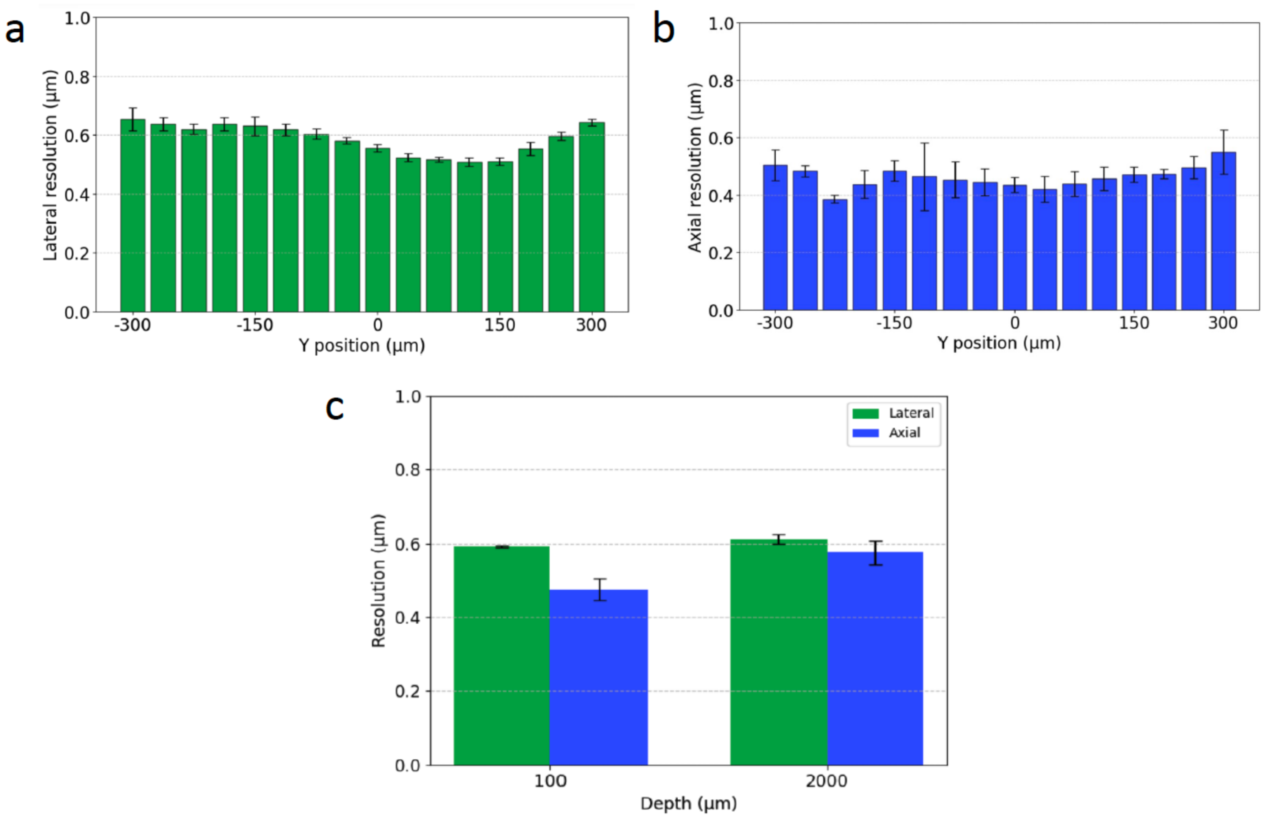

Supplementary Figure 4: Characterization of uniformity of resolution across FOV and volume. a Lateral resolution vs. Y position. **b** Axial resolution vs. Y position with confidence intervals, data averaged over 5 volumes. **c** Resolution at different depths in the sample, as characterized by imaging beads 100 μm and 2000 μm deep in a gel sample.

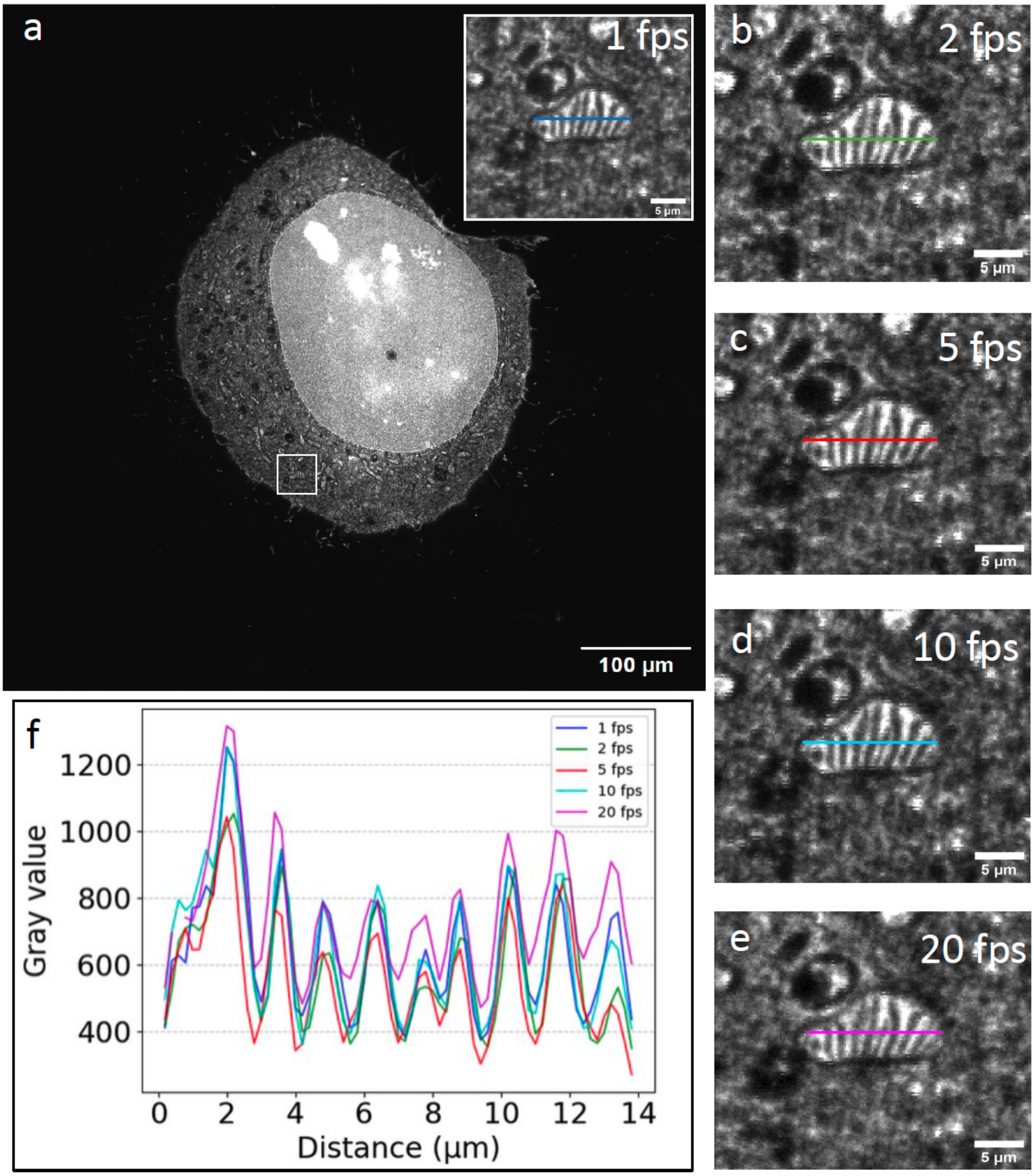

Supplementary Figure 5: Signal variation across frame rates: a Image of a pan-expanded HeLa cell stained with CF568 NHS Ester imaged at 1 fps. Inset shows a zoomed-in view of a single mitochondrion. **b, c, d, e** Zoomed-in views of the same mitochondrion imaged at 2, 5, 10, 20 fps, respectively. The laser power used to acquire these images was adjusted to achieve approximately the same signal levels: 1 fps: 0.51 mW; 2 fps: 1.0 mW; 5 fps: 2.9 mW; 10 fps: 7.7 mW; 20 fps: 51 mW (powers measured in the focal region of the objective). **f** Plot of line profiles across the same mitochondrion indicating comparable signal across frame rates.

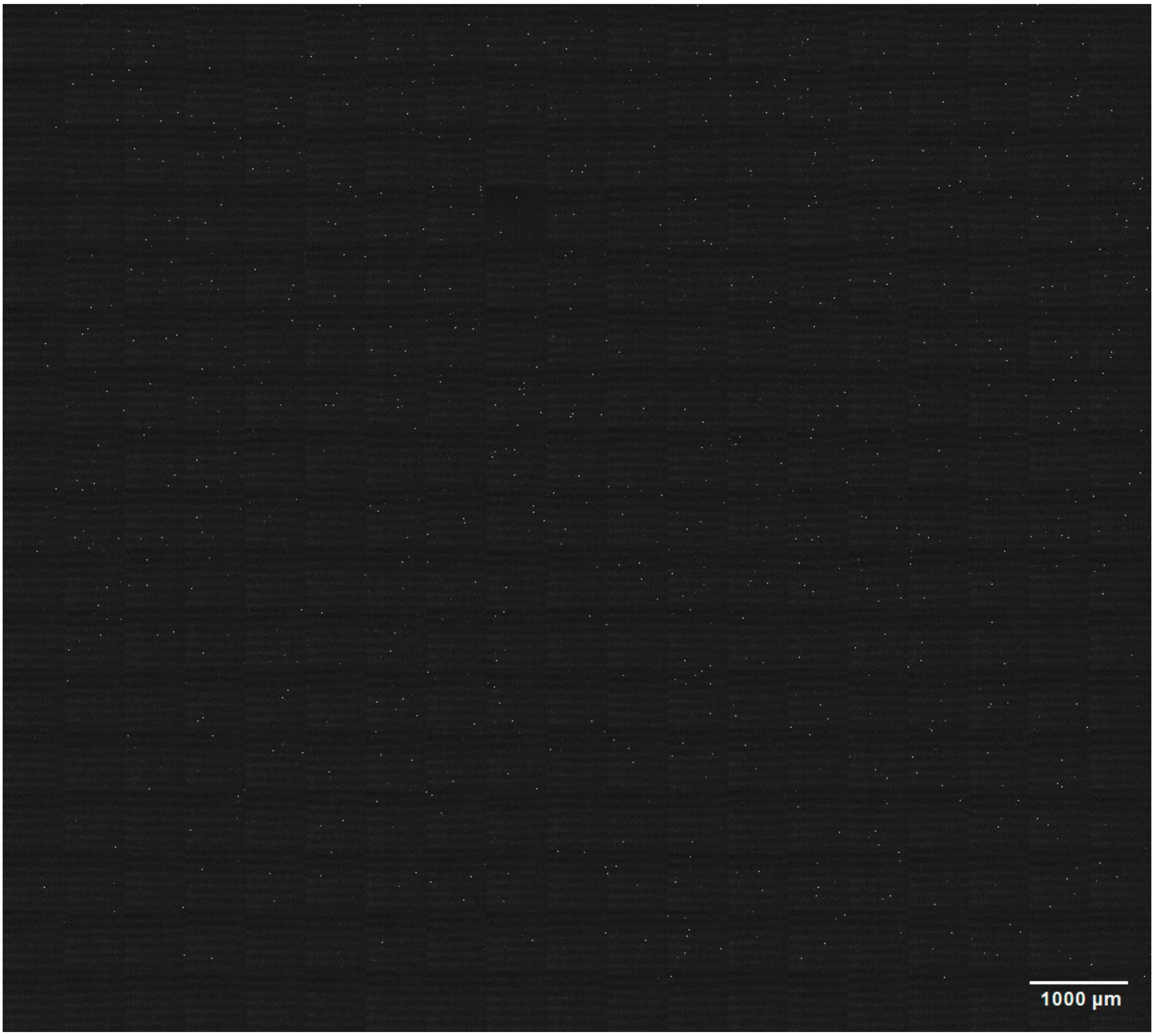

Supplementary Figure 6: Large FOV imaging. 2D tile (10.88 mm x 12.16 mm) of beads embedded in expansion gel.

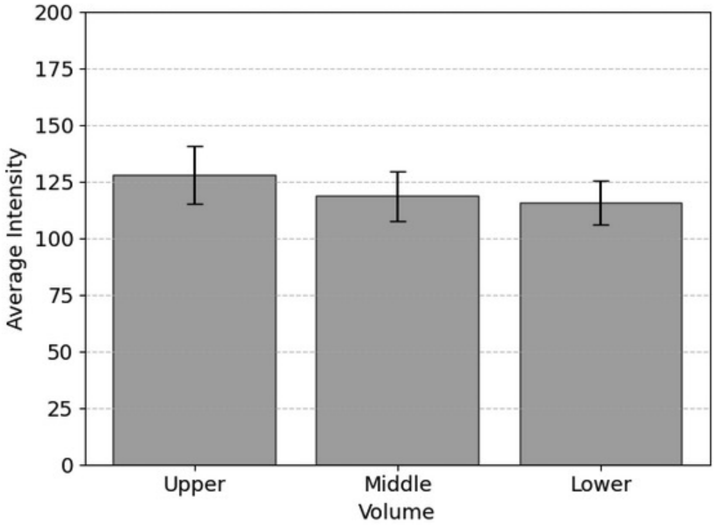

Supplementary Figure 7: Brightness vs. depth plot of a pan-expanded brain tissue sample split into upper, middle, and lower volumes indicating that the decrease in brightness with depth is nearly negligible (~10%).

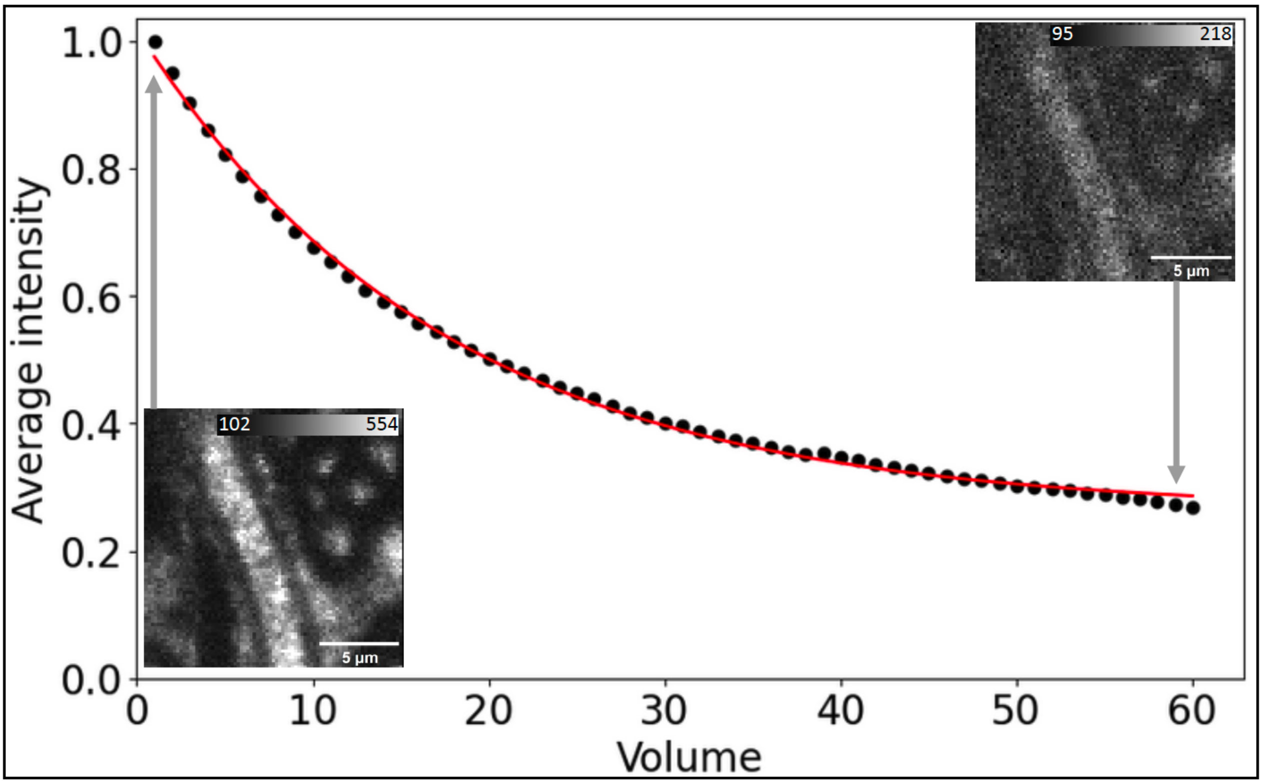

Supplementary Figure 8: Photobleaching characterization. Photobleaching curve of Atto 488 NHS Ester obtained by imaging a pan-expanded brain tissue volume 60 times at 10 fps, Z-step 200 nm, 500 slices. Tau value of fit: 17.36 volumes (~868 sec). Left inset: Zoomed-in view of a representative slice from volume 1. Right inset: Zoomed-in view of a representative slice from volume 59 showing decreased intensity due to photobleaching of Atto 488 NHS Ester.

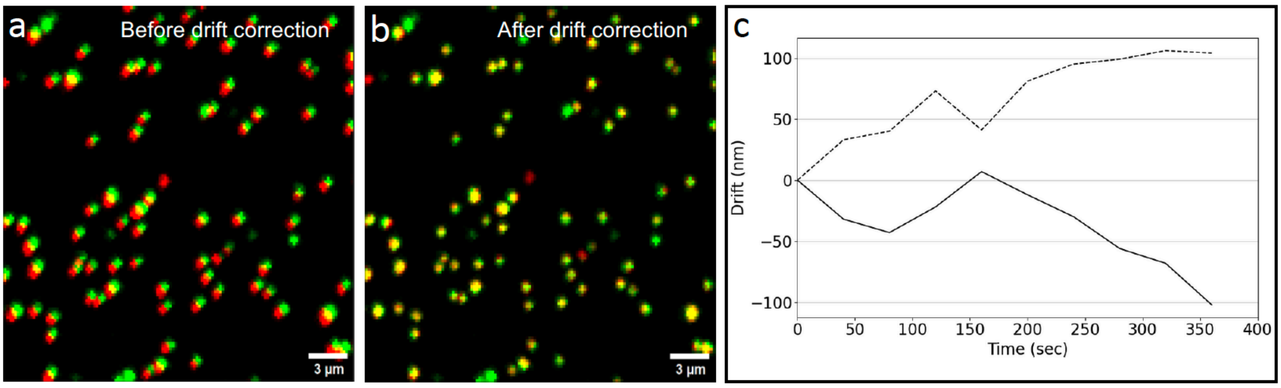

Supplementary Figure 9: Drift characterization. a Zoomed-in view of MIP of beads embedded in agarose merged view of round 1 volume (green) and round 10 volume (red) before and after (**b**) drift correction. **c** Drift vs. time plot of the same bead volume imaged 10 times. We notice a minimal drift of ~100 nm. Using the same dataset, we calculated the RMS error between two sequential volumes and got 9.23 nm and 7.45 nm for X and Y direction, respectively.

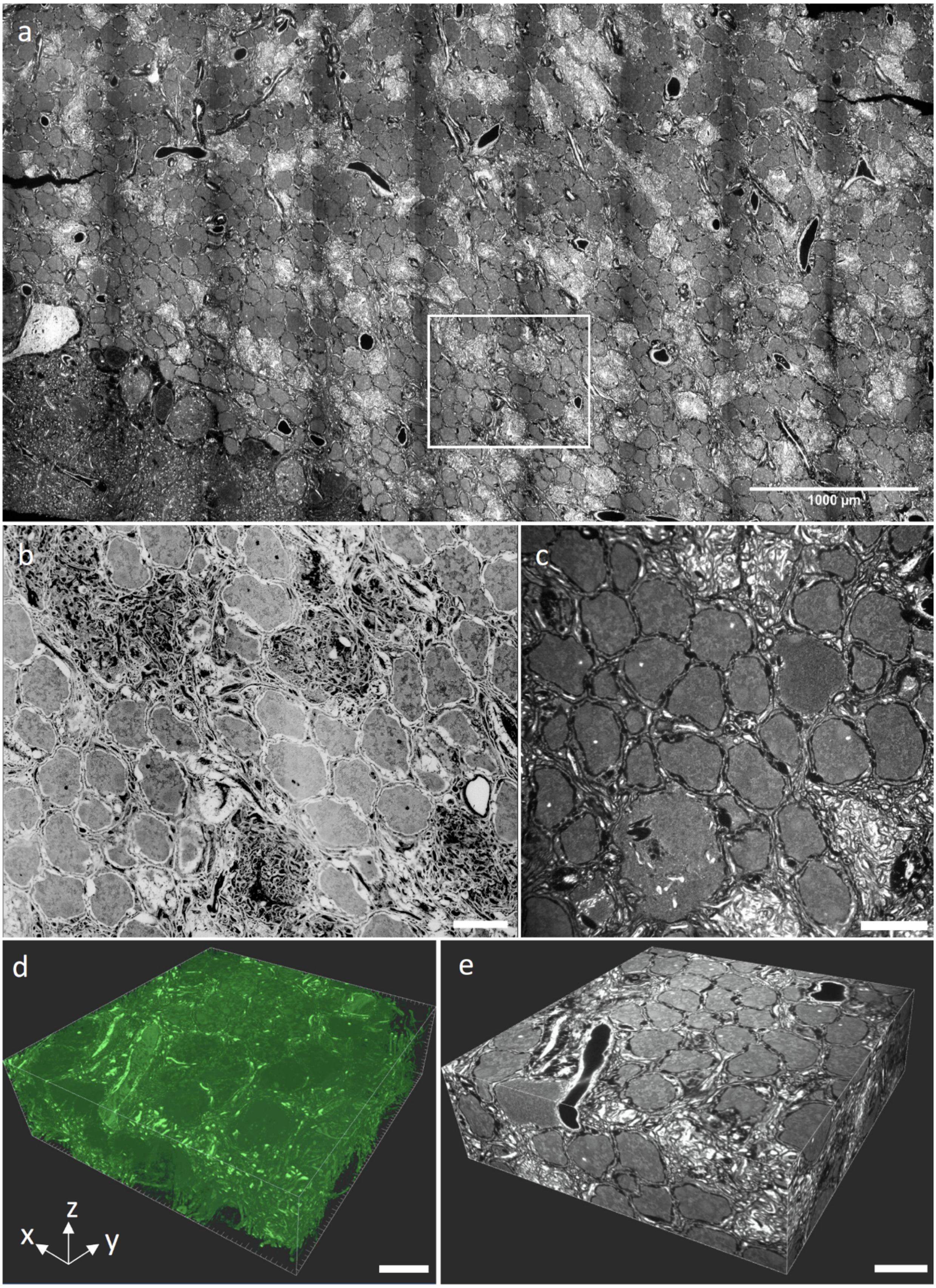

Supplementary Figure 10: Large-FOV imaging of pan-expanded mouse brain tissue. a Tiled pan-ASLM image of a 5.51 mm x 3.07 mm pan-expanded mouse brain tissue section stained with Atto 643 NHS ester. **b** Zoomed-in view of the region shown in (**a**) with inverted color table. **c** A single-FOV image showing pan-expanded brain tissue from the same dataset. **d** 3D rendering of brain tissue volume from the same dataset as (**c**). **e** 3D rendering of brain tissue volume from the same dataset as (**c**). Scale bars are not corrected for the expansion factor. Scale bars: (**b-e**) 100 µm.

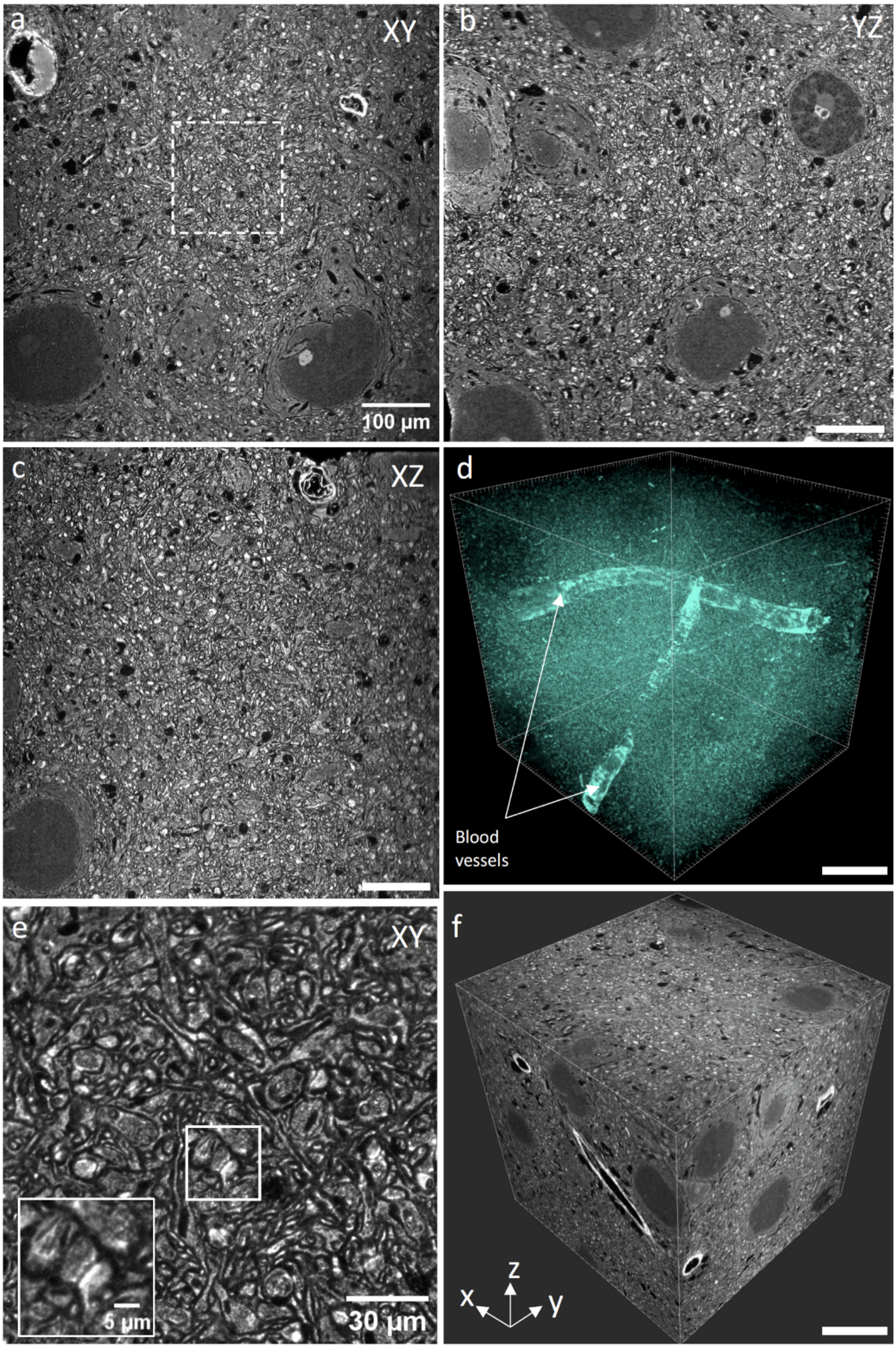

Supplementary Figure 11: pan-ASLM imaging of pan-expanded mouse brain tissue. a XY slice of a single-FOV image stack of a pan-expanded mouse brain tissue section stained with Atto 643 NHS ester. **b, c** YZ view (**b**) and XZ (**c**) of the same region as shown in (**a**). **d** MIP of the brain tissue volume shown in (**a**) revealing blood vessels. **e** Zoomed-in view of the region shown in the box in (**a**). The inset shows the synapse highlighted by the white box in (**e**). **f** 3D rendering of the brain tissue volume shown in (**d**). Scale bars are not corrected for the expansion factor. Scale bars: (**b, c**) 100 µm, (**d**, **f**) 150 µm.

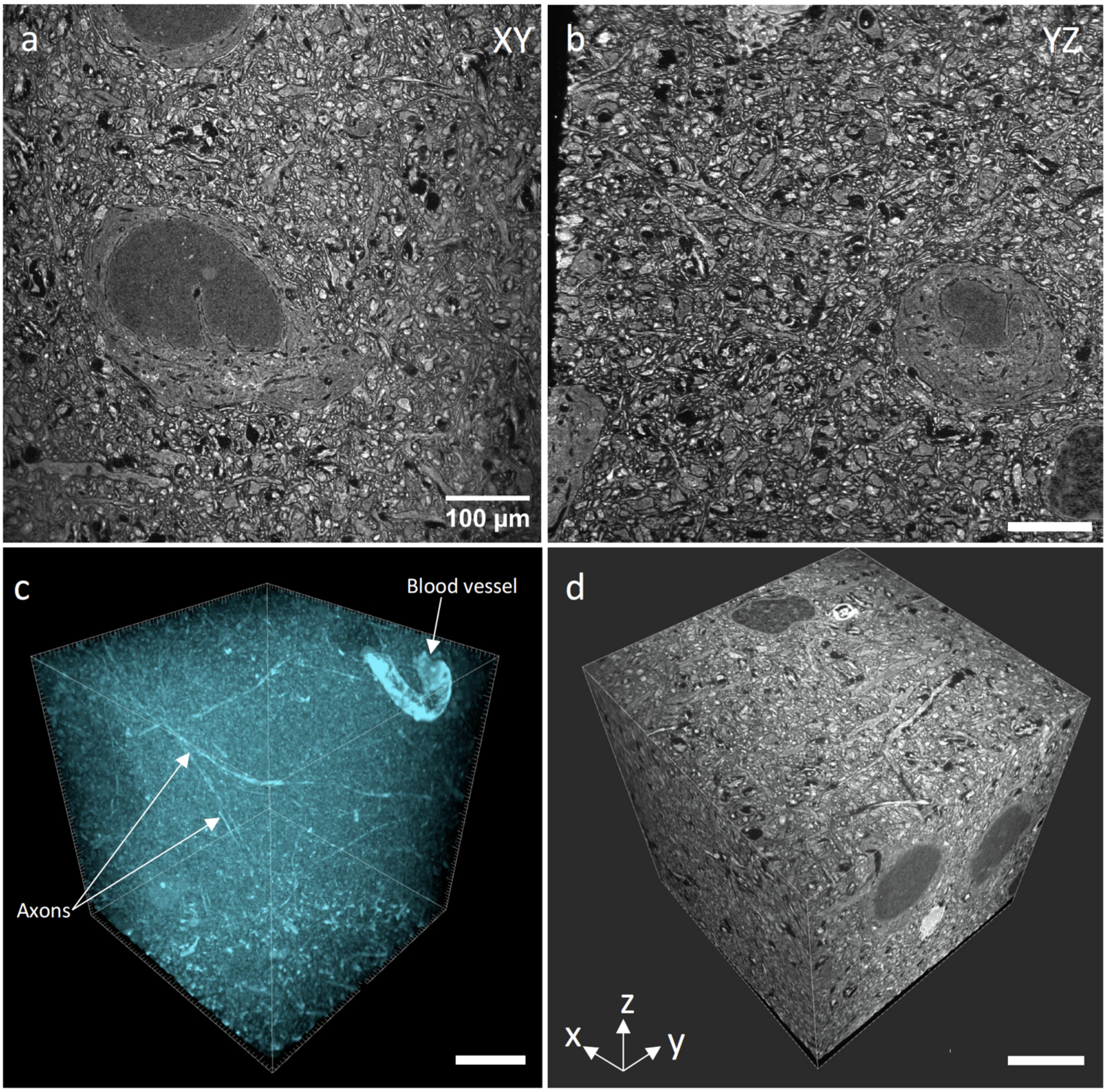

Supplementary Figure 12: pan-ASLM imaging of pan-expanded mouse brain tissue. a XY slice of a single-FOV image stack of a pan-expanded mouse brain tissue section stained with Atto 643 NHS ester. **b** YZ view of the same region as shown in (**a**). **c** MIP of the brain tissue volume shown in (**a**) revealing blood vessels and axons. **d** 3D rendering of the same brain tissue volume. Scale bars are not corrected for the expansion factor. Scale bar: (**b**) 100 µm, (**c**, **d**) 150 µm.

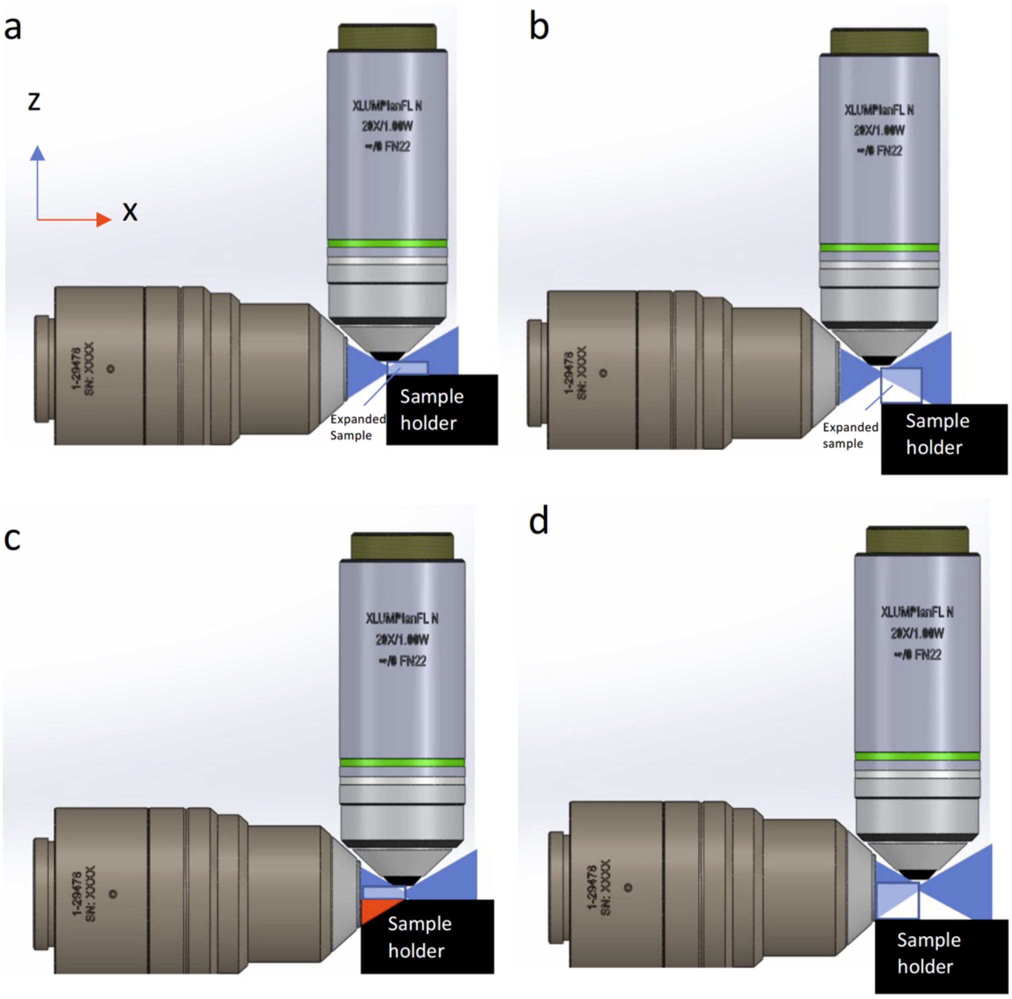

Supplementary Figure 13: pan-ASLM imaging volume and sample thickness. a, b The front of a thin (~2-mm thick) (**a**) and thick (~8 mm) sample (**b**) can be imaged with pan-ASLM across the whole depth. **c, d** When focusing deep into the sample with the illumination objective, the light sheet is clipped by the sample holder for the thin sample (shown in red) (**c**), but not the thick sample (**d**). Using a thick gel even for thin tissue samples or adding a spacer underneath a thin sample prevents this problem.

Components	Company	Part number/ model name	Quantity
Laser(488 nm), 150mW	Coherent	OBIS LX 488nm 150mW	1
Laser(561 nm), 500mW	MPB Communications	2RU-VFL-P-500-561-B1R	1
Laser(595 nm), 500mW	MPB Communications	2RU-VFL-P-500-595-B1R	1
Laser(642 nm), 2W	MPB Communications	2RU-VFL-P-2000-642-B1R	1
Dichroic mirror	Semrock	LM01-613-25	1
Dichroic mirror	Semrock	Di02-R561-25x36	1
Dichroic mirror	Semrock	Di02-R488-25x36	1
AOTFnC-VIS	AA Optoelectronic	AOTFnC-VIS	1
AOTF driver, 8 channel	AA Optoelectronic	MPDS8C-B66-22-74-158	1
Achromatic Half-Wave Plate	Thorlabs	AHWP05M-580	1
f=50 mm Achromat	Thorlabs	AC254-50-A	2
f=300mm, Achromat	Thorlabs	AC254-300-A	1
piShaper	Edmund Optics	#12-644	1
Periscope assembly	Thorlabs	RS99	1
f=150mm, Achromat	Thorlabs	AC254-150-A	1
f=100mm, Cylindrical lens	Thorlabs	ACY254-100-A	1
f=150mm, Achromat	Thorlabs	AC254-150-A	3
Polarizing beam splitter	Thorlabs	CCM1-PBS251/M	1
Quarter wave plate	Thorlabs	AQWP10M-580	1
Remote focusing objective	Evident	UPLXAPO20X/0.8 NA	1
Scan mirror	Thorlabs	BB03-E02	1
Voice coil actuator	Equipment Solutions	LFA-10	1
Servo controller for voice coil	Servo controller	SCA814	1
f=200mm, Achromat	Thorlabs	AC254-50-A	1
Illumination objective	ASI	54-12-8	1
Detection objective	Evident	XLUMPLFLN20XW	1
Piezo objective actuator	Thorlabs	PIA13	1
Multiband emission filter	Chroma	ZET405/488/561/640mv2	1
Magnification changer	Evident	U-CA	1
Tube lens	Evident	SWTLU-C	1
Camera	Teledyne Photometric	Kinetix	1
Vertical breadboard	Thorlabs	MBH4545/M	1
18" Right-angle support	Thorlabs	VB01B/M	2
Coarse stage for sample (X,Y)	Physik Instrumente	V-508.231	2
Sample stage (Z)	Physik Instrumente	L-306.011112	1
ACS controller	Physik Instrumente	G-901.R3197	1
Translation Stage with Standard Micrometer	Thorlabs	MT1/M	2
Base Plate for MT Series Translation Stages	Thorlabs	MT401/M	1
Plastic head thumb screw	McMaster Carr	92605A112	1
Sample chamber	Tristar	Custom	1
Sample holder	Tristar	Custom	1
Cage arm	Tristar	Custom	2
Stage baseplate	Tristar	Custom	1
2nd stage baseplate	Tristar	Custom	1
3rd stage baseplate	Tristar	Custom	1
Crossbar	Tristar	Custom	1
Sample chamber side mount	Tristar	Custom	1
Sample chamber upper mount	Tristar	Custom	1
Laser head mount	Tristar	Custom	3
AOTF mount	Tristar	Custom	1

Supplementary Table 1: pan-ASLM parts list.

Frame rate (fps)	Acquisition time (ms)	Flyback (ms)	Total time (ms)	2 pixels exposure time (ms)	4 pixels exposure time (ms)	8 pixels exposure time (ms)
1	976	24	1000	0.61	1.22	2.44
2	476	24	500	0.2975	0.595	1.19
2.5	376	24	400	0.235	0.47	0.94
5	176	24	200	0.11	0.22	0.44
10	76	24	100	0.0475	0.095	0.19
12.5	46	24	70	0.02875	0.0575	0.115
20	26	24	50	0.01625	0.0325	0.065

Supplementary Table 2: Exposure times for different frame rates and rolling shutter widths (2 pixels, 4 pixels, 8 pixels).

Captions of Supplementary Movies

Supplementary Movie 1: A Z-stack of a zoomed-in view of a pan-expanded HeLa cell with NHS Ester pan-staining shown in Figure 2.

Supplementary Movie 2: 3D rendering of a pan-expanded HeLa cell stained with Sytox Green and NHS Ester pan-staining shown in Figure 4a.

Supplementary Movie 3: 3D rendering of a pan-expanded mouse kidney tissue of the proximal tubule showing a zoomed-in view of the brush borders.

Supplementary Movie 4: 3D rendering of a pan-expanded mouse kidney tissue of the glomerulus showing zoomed-in views of podocyte foot processes.

Supplementary Movie 5: Fly-through video of a tiled 6.11 mm x 3.07 mm area of a pan-expanded mouse cortex with NHS Ester pan-staining shown in Figure 6a.
